# Urinary Extracellular Vesicles: Potential Biomarkers of Renal Function in Diabetic Patients

**DOI:** 10.1155/2016/5741518

**Published:** 2016-12-25

**Authors:** Agnieszka Kamińska, Mark Platt, Joanna Kasprzyk, Beata Kuśnierz-Cabala, Agnieszka Gala-Błądzińska, Olga Woźnicka, Benedykt R. Jany, Franciszek Krok, Wojciech Piekoszewski, Marek Kuźniewski, Ewa Ł. Stępień

**Affiliations:** ^1^Department of Medical Physics, Marian Smoluchowski Institute of Physics, Faculty of Physics, Astronomy and Applied Computer Science, Jagiellonian University, 30-348 Kraków, Poland; ^2^Department of Chemistry, Loughborough University, Loughborough LE11 3TU, UK; ^3^Laboratory of High Resolution Mass Spectrometry, Regional Laboratory of Physicochemical Analysis and Structural Research, Faculty of Chemistry, Jagiellonian University, 30-060 Kraków, Poland; ^4^Department of Diagnostics, Chair of Clinical Biochemistry, Jagiellonian University Medical College, 31-501 Kraków, Poland; ^5^St' Queen Jadwiga Clinical District Hospital No. 2, 35-301 Rzeszów, Poland; ^6^Department of Cell Biology and Imaging, Institute of Zoology, Faculty of Biology and Earth Sciences, Jagiellonian University, 30-387 Kraków, Poland; ^7^Department of Solid State Physics, Marian Smoluchowski Institute of Physics, Faculty of Physics, Astronomy and Applied Computer Science, Jagiellonian University, 30-348 Kraków, Poland; ^8^Department of Analytical Chemistry, Faculty of Chemistry, Jagiellonian University, 30-060 Kraków, Poland; ^9^Department of Nephrology, Jagiellonian University Medical College, 31-501 Kraków, Poland

## Abstract

The aim of this study was to check the relationship between the density of urinary EVs, their size distribution, and the progress of early renal damage in type 2 diabetic patients (DMt2). Patients were enrolled to this study, and glycated hemoglobin (HbA1c) below 7% was a threshold for properly controlled diabetic patients (CD) and poorly controlled diabetic patients (UD). Patients were further divided into two groups: diabetic patients without renal failure (NRF) and with renal failure (RF) according to the Glomerular Filtration Rate. Density and diameter of EVs were determined by Tunable Resistive Pulse Sensing. Additionally, EVs were visualized by means of Transmission and Environmental Scanning Electron Microscopy. Nano-liquid chromatography coupled offline with mass spectrometry (MALDI-TOF-MS/MS) was applied for proteomic analysis. RF had reduced density of EVs compared to NRF. The size distribution study showed that CD had larger EVs (mode) than UD (115 versus 109 nm; *p* < 0.05); nevertheless the mean EVs diameter was smaller in controls than in the CD group (123 versus 134 nm; *p* < 0.05). It was demonstrated that EVs are abundant in urine. Albumin, uromodulin, and number of unique proteins related to cell stress and secretion were detected in the EVs fraction. Density and size of urinary EVs reflect deteriorated renal function and can be considered as potential renal damage biomarkers.

## 1. Introduction

Recently, the incidence of diabetes mellitus has grown significantly throughout the world and diabetes becomes the most common cause of kidney injury. It is supposed that about 30 percent of patients with diabetes of type 1 (DMt1) and 10 to 40 percent of those with type 2 (DMt2) will suffer from renal damage [1–3]. Most of cells release small membrane spherical structures called extracellular vesicles (EVs) which can be classified into three groups: exosomes (50–100 nm), microvesicles (100–1000 nm), and apoptotic bodies. These vesicles differ in their composition and subcellular origin. EVs can be found in several body fluids, including plasma, urine, saliva, and milk [4]. In particular, urine is a rich reservoir of these vesicles which originate from the cells facing the urinary lumen (epithelial cells). The urinary EVs can reflect the state of the damage of the kidney. Results of several studies indicate that EVs originating from urine have recently emerged as an interesting source of diagnostic disease biomarkers and contain molecules involved in intercellular communication [5–9]. Changes in excretion rates of specific proteins also can have predictive value in the early diagnosis of renal damage [10].

Existing clinical markers such as serum creatinine or urine albumin level are not very sensitive and are generally increased when acute or chronic renal injury is well established [11]. Reliable biomarkers of renal injury are lacking in the renal care. Creatinine measured by laboratories provides little information about the underlying cause of renal injuries and is less accurate for patients with low muscle mass [12, 13]. In diabetes, the most serious and life treating complication is diabetic nephropathy. To avoid this end stage complication there is a growing need to discover novel noninvasive biomarkers of primary renal damage which allow detecting changes in kidney at early stage [14]. In the present study we test the hypothesis that the density and size of urinary EVs can be considered as biomarkers of renal damage in DMt2 patients.

The motivation of this study was to demonstrate the potential usefulness of urinary EVs in diagnostics of early renal failure as a complication of diabetes. In order to achieve this goal we applied the modern approach for urine analysis: Tunable Resistive Pulse Sensing (TRPS) for EVs enumeration and size distribution analysis, a nano-liquid chromatography technique coupled offline with mass spectrometry (MALDI-TOF-MS/MS) for proteomic analysis and electron microscopy (Transmission Electron Microscopy (TEM); Environmental Scanning Electron Microscopy (ESEM)) for EVs visualization.

## 2. Materials and Methods

### 2.1. Study Group

Sixty patients (20 women and 40 men) with type 2 diabetes mellitus (DMt2) were enrolled to the present study. These patients were divided into groups: CD, properly controlled (*n* = 24), and UD, poorly controlled diabetes (*n* = 36). As a control, ten healthy subjects (4 women and 6 men) with an average age of 52 (SD = 7) years were included. The studied groups were allocated according to the criterion of glycated hemoglobin (HbA1c) levels. According to Polish Diabetes Association guidelines from 2014, a HbA1c level of 7% is general criterion of carbohydrate metabolism compensation. Patients in whom HbA1c levels exceed 7% are considered as they have poorly controlled diabetes. What is more, diabetic patients were further classified into two groups: diabetic patients without renal failure (NRF) and with renal failure (RF). A selection of RF was Glomerular Filtration Rate (GFR) below 60 mL/min/1.73 m^2^ from MDRD2 formula. Microalbuminuria was defined as 20–200 mg/L and macroalbuminuria >200 mg/L albumin filtration. The clinical characteristics of the studied groups are presented in Tables 1 and 2.

### 2.2. Urine Samples Collection and Preparation

First morning urine specimens were collected into sterile containers (F.L. Medical SRL, Torreglia, Italy) from diabetic patients and healthy subjects. Typically 50 mL first void urine was used for the isolation of the urinary extracellular vesicles and processed within 2 h of collection. Samples were centrifuged in a Hermle Z300K (Hermle Labortechnik GmbH, Wehingem, Germany) for 10 min in 3000*g* at 4°C to remove cells and larger debris. After this step supernatants were aliquoted and frozen at −80°C for further analysis. Immediately before the TRPS measurement, samples were thawed in a water bath at 37°C and then vortexed for 30 s, diluted 1 : 1 in PBS (Cat. number P4417, Sigma-Aldrich, St. Louis, USA), vortexed for 10 s, and used for analysis. For mass spectrometry and electron microscopy analysis, supernatants were ultracentrifuged in 150 000*g* for 1 h at 4°C (Optima™ MAX-XP, Beckman Coulter Life Sciences, Indianapolis, USA) using a horizontal rotor (Cat. number 367280, MLS-50 Swinging-Bucked Rotor, Beckman Coulter Life Sciences, Indianapolis, USA).

### 2.3. Blood Samples

Blood samples for biochemical and hematology analysis were drawn by venipuncture of the antecubital vein using a 21-gauge needle and the Sarstedt S-Monovette blood collection system (Sarstedt AG & Co., Nümbrecht, Germany) following application of a light tourniquet. For complete blood count analysis and HbA1c levels, EDTA anticoagulant was used. For biochemical analysis, blood was collected in serum separator tubes. Standard blood tests were performed by means of the hematology analyzer (ELITech Group, Puteaux, France). HbA1c level was measured on D-10 analyzer (D-10 hemoglobin testing system, Bio-Rad Laboratories Inc., California, USA).

### 2.4. Tunable Resistive Pulse Sensing Technology

The size and density of urinary EVs were determined by Tunable Resistive Pulse Sensing (TRPS) technique using qNano system and tunable pore specimen, NP150 from Izon Science (Izon Science Ltd., Christchurch, New Zealand). Principles of the technique were described in [15–18]. To detect particles in the range 60–480 nm the pores labeled NP150 were used. Polystyrene beads of known raw concentration (1.5*E* + 13/mL) and diameter of 105 nm were sourced from Izon Science and were used as a calibrant. Typically a bandwidth filter of 5 kHz was applied during measurements. For the electrolyte and dilution buffer we used PBS. In all measurements 75 *μ*L of electrolyte buffer was placed in the lower fluid cell and the volume in the upper fluid cell was 40 *μ*L. Each sample was measured in triplicate. The density, mean, and mode diameter of EVs are expressed as median (IQR). Data capture was performed using Izon's control suite 3.1 software.

### 2.5. Proteomics (Nano-LC-MALDI-TOF/TOF Mass Spectrometry)

For proteomics analysis urinary EVs were isolated from microalbuminuric (CD) and macroalbuminuric (UD) patients and healthy subjects, at least *n* = 3 from each group. After ultracentrifugation, urine supernatants (6 mL) were used for analysis. Obtained pellet was resuspended in 60 *μ*L 10% SDS (Cat. number L3771, Sigma-Aldrich, St. Louis, USA), 10 *μ*L  1M TRIS (Cat. number T1503 Sigma-Aldrich, St. Louis, USA), and 30 *μ*L deionized water [19]. Protein concentration was determined using BCA method (Cat. number 23227, Pierce Biotechnology, Thermo Scientific, USA). Mean protein concentration was 1.14 ± 1.04 mg/mL; the total protein amount used for MS was 40 *μ*g. Proteomic analysis was performed by means of a nano-liquid chromatograph (EASY-nLC II™, Bruker Daltonics, Germany). The detailed methodology was previously published [20]. The precision tolerance was 100 ppm for peptide masses and 0.7 Da for fragment ion masses. Individual peptide matches with scores above 28 were considered statistically significant. Proteins identification was performed manually, based on two unique peptides with the probability less than 0.05. The protein classification was performed by means of a free algorithm applied in the PANTHER Classification System (Version 11.0, released July 15, 2016) [21]. The analysis of overlapping proteins within healthy subjects, CD, and UD was performed by a tree-circle Venn diagram software [22].

### 2.6. Transmission Electron Microscopy and Environmental Scanning Electron Microscopy

#### 2.6.1. ESEM

Urine sample from a healthy donor (100 mL) was centrifuged in 3000*g* and next supernatant was ultracentrifuged in 150 000*g* for 1 h at 4°C. EVs pellet was resuspended in 60 *μ*L of PBS and 20 *μ*L of EVs solution was placed on 1 × 1 cm poly-l-lysine slide (Cat. number J2800 AMNZ, Thermo Fisher Scientific, Waltham, Massachusetts, USA) and incubated for 1 h in humid chamber at RT. After incubation the slide was washed twice in PBS and fixed in 3.7% glutaraldehyde in PBS for 30 min followed by salt removal stage. The slide with EVs was washed with two aqueous PBS dilutions, 50% PBS, 25% PBS, and deionized water, each for 1 minute. Next, the dehydration was applied by immersing sample for 30 seconds in ethyl alcohol solutions as follows: 10%, 20%, 30%, 40%, 50%, 60%, 70%, 80%, 90%, and absolute ethanol. Afterwards, sample was dried for 24 h under cover at RT [23].

The Environmental Scanning Electron Microscopy (ESEM) measurements were performed using SEM Quanta 3D FEG microscope by FEI Company (USA) operated at Institute of Physics Jagiellonian University, Kraków, Poland. The ESEM images were collected by GSED detector using electrons of 5 keV energy. During measurements the specimen was kept at 100 Pa of water vapor at RT.

#### 2.6.2. TEM

Two urine samples from a healthy donor and one UD were prepared in the same way as for ESEM analysis. Samples were centrifuged in Eppendorf tube and fixed with 2.5% glutaraldehyde (Cat. number G5882, Sigma-Aldrich, St. Louis, USA) in 0.1M cacodylic buffer (Cat. number C4945, Aldrich, St. Louis, USA) for 2 h at RT and then postfixed in 1% osmium tetroxide solution (1 hour). Samples were dehydrated by passing through a graded ethanol series and embedded in PolyBed 812 at 68°C.

Ultrathin sections were collected on 300 mesh grids or one slot made from copper. Additionally the latter was covered with formvar film. Next the sections were contrasted using uranyl acetate and lead citrate. For observation the electron microscopy from JEOL company JEOL JEM 2100HT (Jeol Ltd, Tokyo, Japan) was used at accelerating voltage 80 kV.

### 2.7. CD81 TRIFIc Exosome Assay

Europium Time Resolved Fluorescence assay, Cat. number EX103 (Cell Guidance Systems Ltd., Cambridge, United Kingdom), was used to measure abundance of human CD81 protein in the surface of exosomes in the same urine samples. In the TRIFIc exosome assay the same antibody is used for binding of target to the assay plate and for detection. This assay consists of a monoclonal antibody (labeled with biotin) bound to streptavidin coated plate that captures proteins which are present in the surface of exosomes. An identical monoclonal antibody (labeled with Europium) is used for detection. Europium provides a high degree of sensitivity for the assay. For fluorescence detection we used infinite M200 PRO plate reader (Tecan Group Ltd., Männedorf, Switzerland).

### 2.8. Statistical Analysis

Statistica 12 (Dell Statistica, Tulsa, USA) and OriginPro 2016 (OriginLab Corporation, Northampton, USA) were used for statistical analyses and plots design. The distribution of continuous data was verified with Shapiro-Wilk normality test. Results are expressed as mean (SD) for data with normal distribution or median and interquartile ranges (Q1–Q3) for data with not normal distribution. Biochemical and epidemiological data were analyzed by one-way analysis of variance ANOVA or Kruskal-Wallis for comparison among groups. Differences between subgroups were tested with Tukey's post hoc test or Dunn's multiple comparison test. The Mann–Whitney* U* test was used to compare differences between two independent groups. Correlations between EVs density and biochemical parameters were calculated with Spearman's rank correlation test, and multiple regression (backward stepwise regression) was performed to predict the effect of age on the other variables. For all analyses *p* values < 0.05 were considered significant.

### 2.9. Ethical Considerations

This study was approved by The Bioethical Committee of Jagiellonian University in Kraków on 24 October 2013 which accepted all project's protocols and forms, including an information for patients form and a consent form. The permission number KBET/206/B/2013 is valid until 31 December 2016.

## 3. Results

A comparison of biochemical parameters such as serum glucose, urine albumin, urine creatinine, serum creatinine, GFR, EVs density, EVs mode, and mean diameter in CD, UD, and the control group was provided in Table 1. Properly controlled diabetic patients (CD) and poorly controlled diabetic patients (UD) had significantly higher levels of serum glucose (6.8 versus 5.2 mmol/L; *p* < 0.0001 and 9 versus 5.2 mmol/L; *p* < 0.0001) and lower urine creatinine concentration (5 versus 12 mmol/L; *p* = 0.003 and 7 versus 12 mmol/L; *p* = 0.004) in comparison with the control group.

Our results showed statistically significant difference in serum glucose (6.8 versus 9 mmol/L; *p* = 0.0001) and urine albumin (6 versus 37 mg/L; *p* = 0.002) between CD and UD groups. No significant difference was found in serum creatinine concentration, GFR and EVs density between these groups. Size distribution analysis showed that CD had significantly larger EVs mode diameter besides UD (115 versus 109 nm; *p* = 0.031). The mean EVs diameter was smaller in controls than in the CD group (123 versus 134 nm; *p* = 0.004).

A comparison of biochemical parameters in RF, NRF, and the control group is provided in Table 2. Compared with the control group, RF had significantly higher levels of serum glucose (8.7 versus 5.2 mmol/L; *p* < 0.0001) and serum creatinine (119 versus 72 *μ*mol/L; *p* < 0.0001) and lower urine creatinine concentration (6 versus 15 mmol/L; *p* = 0.002) and GFR (49 versus 87 mL/min/1.73 m^2^; *p* < 0.0001). NRF had significantly higher levels of serum glucose (7.9 versus 5.2 mmol/L; *p* < 0.0001) and lower urine creatinine concentration (8 versus 15 mmol/L; *p* = 0.003) in comparison with the healthy subjects.

The obtained results indicate that RF had significantly reduced density of EVs compared to NRF (2.57*E*10 versus 8.73*E*10 number/mL; *p* = 0.017). We observed statistically significant difference in serum creatinine (119 versus 73 *μ*mol/L; *p* < 0.0001) and GFR (49 versus 89 mL/min/1.73 m^2^; *p* < 0.0001) between RF and NRF groups and in albumin level between RF and healthy subjects (51 versus 6.2 mg/L; *p* = 0.02). Because of high variability within patients groups, no significant difference was found between EVs mode diameters between RF and NRF.

Results of Spearman's* rho* test for relationship between EVs density and biochemical parameters are presented in Table 3. We observed a negative tendency between EVs density and serum glucose level in UD (*R* = −0.33) and negative correlation in RF (*R* = −0.66) patients (Figure 2). There was no correlation between these parameters in CD (*p* = 0.19) and NRF (*p* = 0.16). We found positive relationship between EVs density and urine creatinine concentration in CD (*R* = 0.52) and NRF (*R* = 0.33) (Figure 3). There was no correlation between these parameters in UD (*p* = 0.87) and RF (*p* = 0.08).

Taking into consideration that the age can influence renal function, multiple regression (backward stepwise regression) was performed to show the impact of age on changes in the amount of EVs (see Supplementary Table 1 in the Supplementary Material available online at http://dx.doi.org/10.1155/2016/5741518). Additionally, the correlations of specific biochemical parameters (creatinine, albumin, serum glucose, etc.) with age have been analyzed. Not surprisingly, there was no correlation in control group. The age related negative relationship was observed in CD and NRF group in terms of creatinine clearance (GFR). Such relationship was less significant in patients with more advanced stage of disease (Supplementary Table 2).

**Figure 1:**
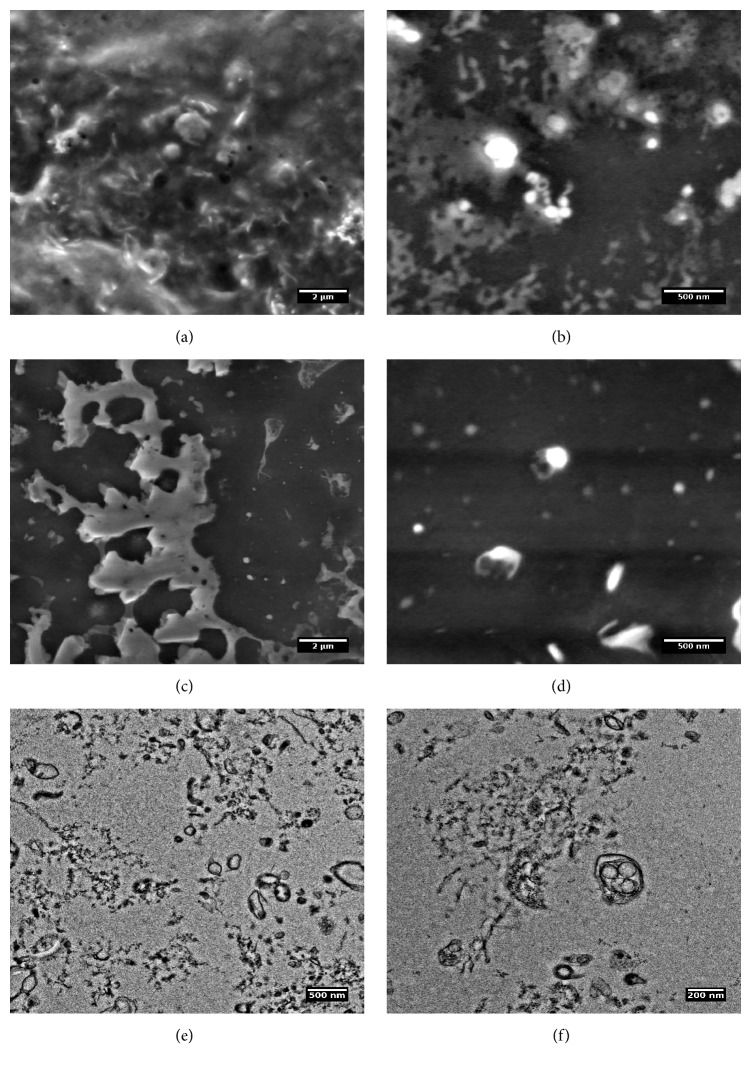
Environmental Scanning Electron Microscopy (ESEM) (a–d) and Transmission Electron Microscopy (e, f) images of urinary extracellular vesicles (EVs) isolated from a urine sample. ESEM images show that EVs form aggregates and they are clustered on the surface. TEM analysis visualizes the variety of different vesicle-like objects in diameter mostly around 130–160 nm. Interestingly, multivesicle objects were also present in urine that confirms integrity of EVs during preparation.

**Figure 2:**
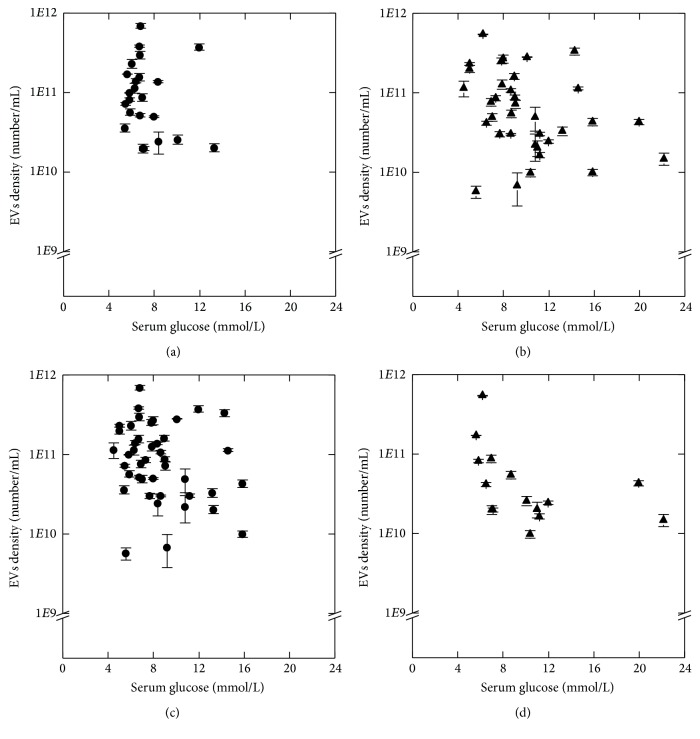
Relationship between EVs density and serum glucose level in study groups: CD (a), UD (b), NRF (c), and RF (d). EVs density values are given as mean (SD). Spearman's rank correlation coefficient, *p* < 0.05.

**Figure 3:**
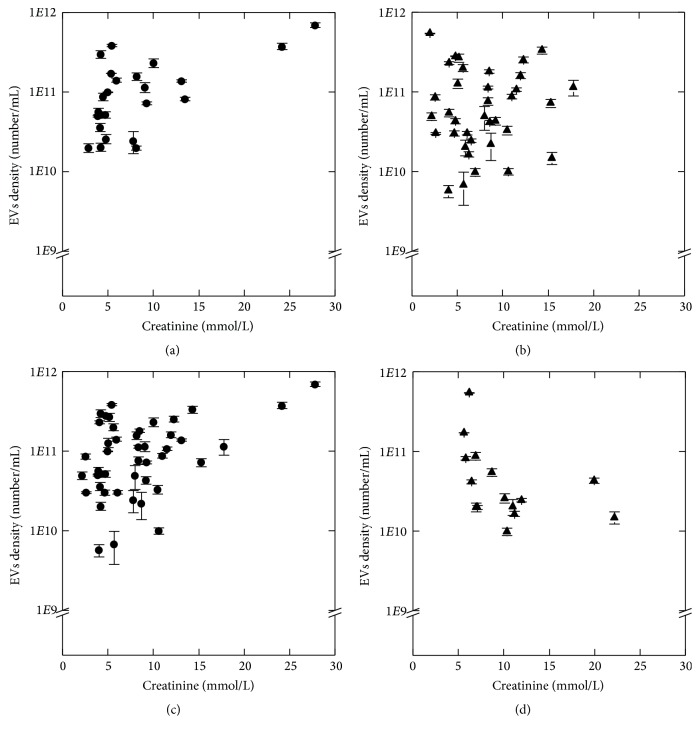
Relationship between EVs density and urine creatinine concentration in study groups: CD (a), UD (b), NRF (c), and RF (d). EVs density values are given as mean (SD). Spearman's rank correlation coefficient, *p* < 0.05.

Environmental Scanning Electron Microscopy (ESEM) confirmed the presence of the EVs in pellets sedimented after ultracentrifugation of collected samples (Figures 1(a)–1(d)). Washed EVs formed clustered aggregates, which were better distinguishable by means of TEM (Figures 1(e) and 1(f)). The size of EVs was estimated in the range of 130–160 nm. However, a number of smaller and bigger vesicles and other objects were observed.

**Figure 4:**
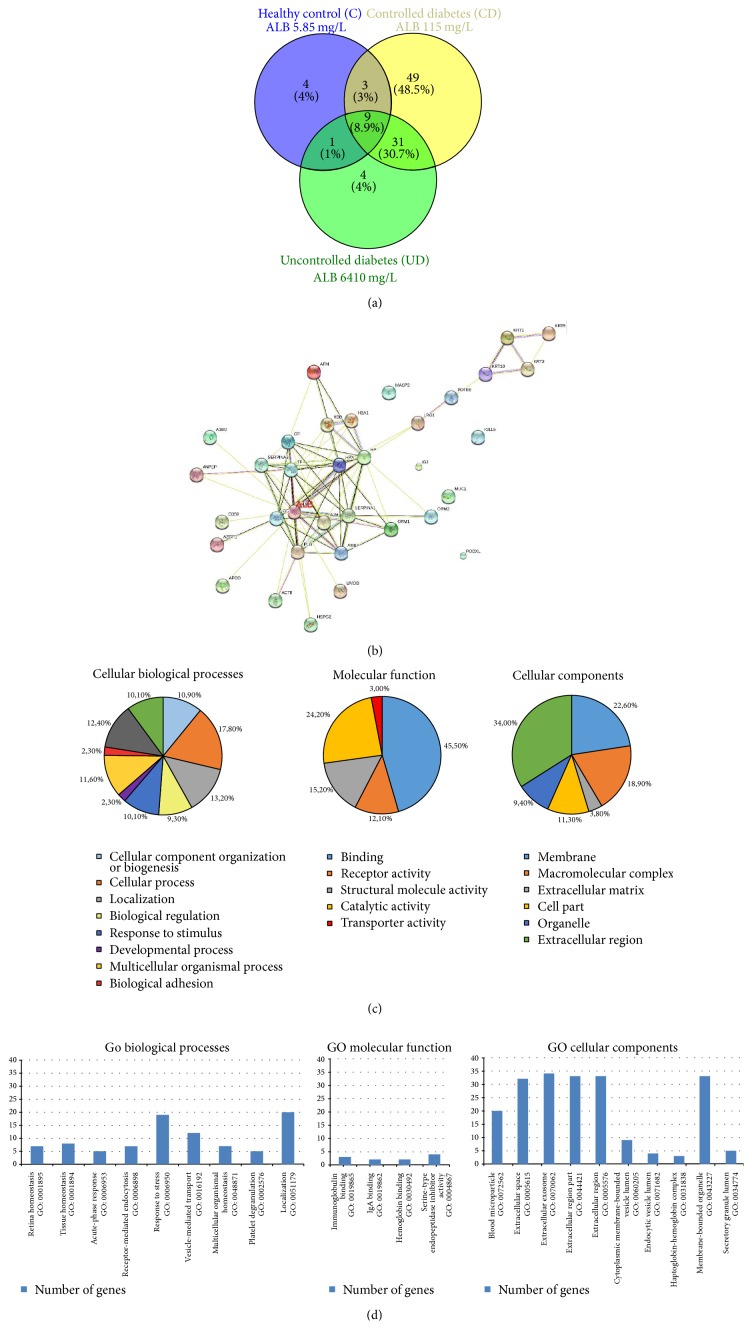
Proteomic analysis of urinary extracellular vesicles: mass spectrometry results from representative samples. (a) Venn diagram shows that in EVs from controlled diabetic patient with microalbuminuria there is a large number of unique proteins (*n* = 49), in a healthy control and in an uncontrolled diabetic patient with macroalbuminuria the number of unique proteins is very low (*n* = 4) [20]. (b) Protein-to-protein interaction analysis of common 45 proteins selected from Venn diagram shows the central role of albumin among urinary EV-related proteins; the list of submitted proteins is available in a supplementary data file (Supplementary Table 2) [22]. (c, d) Gene Ontology analysis showed that most of identified proteins are related to extracellular region or they are related to membrane organelles (exosomes); their localization corresponds with molecular function (receptors and transport proteins) [19]. A1BG: alpha-1-B glycoprotein; A2M: alpha-2-macroglobulin; ACTB: actin, beta; AFM: afamin; ALB: albumin; AMBP: alpha-1-microglobulin/bikunin precursor; ANPEP: alanyl (membrane) aminopeptidase; APOD: apolipoprotein D; AZGP1: alpha-2-glycoprotein 1, zinc-binding; CD59: CD59 molecule, complement; regulatory protein; CP: ceruloplasmin; GC: group-specific component (vitamin D binding protein); HBA1: hemoglobin, alpha 1; HBB: hemoglobin, beta; HP: haptoglobin; HPX: hemopexin; HSPG2: heparan sulfate proteoglycan 2; IGJ: immunoglobulin J polypeptide; IGLL5: immunoglobulin lambda-like polypeptide 5; KRT1: keratin 1; KRT2: keratin 2; KRT9: keratin 9; KRT10: keratin 10; LRG1: leucine-rich alpha-2-glycoprotein 1; MASP2: mannan-binding lectin serine peptidase 2; MUC1: mucin 1; ORM1: orosomucoid 1; ORM2: orosomucoid 2; PLG: plasminogen; PODXL: podocalyxin-like; POTEE: POTE ankyrin domain family member E; SERPINA1: serpin peptidase inhibitor, clade A, member 1; SERPINA3: serpin peptidase; inhibitor, clade A, member 3; TF: transferrin; UMOD: uromodulin.

In order to see the origin and biological activity of analyzed EVs, the proteomic analysis of a urinary EVs fraction was performed. Despite the fact that urine samples were obtained from patients in different stage of DM and different albuminuria levels, the albumin was the main and most abundant protein detected using mass spectrometry methods (Figures 4(a) and 4(b)). The second abundant protein in urine was uromodulin. Venn analysis shows the possible relationships in a protein profile between CD and UD compared to a control subject. Among total 92 proteins in CD, 49 were unique and 31 were common in CD and UD (Supplementary Table 1). In the UD sample, the total number of proteins was 45 while in the control sample 17 proteins were found. The list of unique proteins for every group was listed in Supplementary Table2. For prediction of common protein interactions the list of common 45 proteins was analyzed by means of Search Tool for the Retrieval of Interacting Genes/Proteins (STRING) [24] (Figure 4(b)). This analysis revealed the central role of albumin in EVs fraction, nevertheless stress related proteins (ceruloplasmin, transferrin) and cellular components (mostly exosome and extracellular region) (Figures 4(c) and 4(d)).

**Figure 5:**
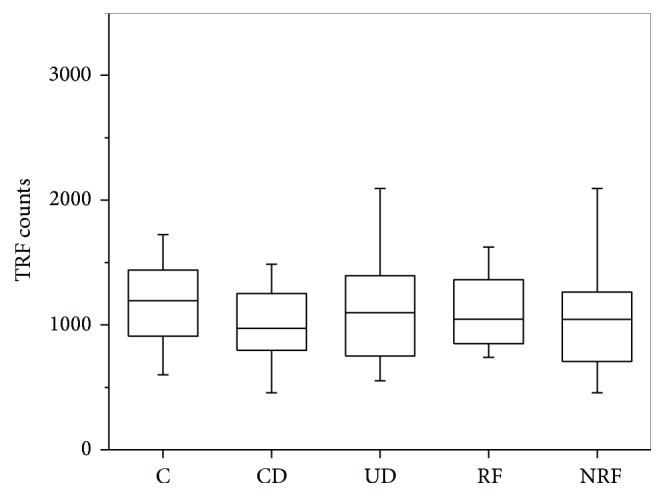
Urine CD81 level. Results from Time Resolved Fluorescence assay. Kruskal-Wallis test: median TRF counts, *p* = 0.5 at *α* < 0.05 significance level.

CD81 TRIFIc exosome assay has not shown any statistical significant differences in CD81 level between study groups, what is presented in Figure 5.

## 4. Discussion

To date, there are no noninvasive methods to characterize renal structural pathophysiological changes [25]. Moreover, biochemical markers are not sensitive enough to characterize the risk of progression of nephropathy and other DM-related complications [13].

In this study we sought to test if the density and size of urinary EVs can be considered as potential biomarkers of early renal damage in DMt2 patients which can lead to diabetic nephropathy. Additionally, we studied if there is any correlation between EVs density and biochemical parameters in diabetic patients and healthy control group.

Our results indicate that diabetic patients with renal failure (RF) had lower density of EVs compared to diabetic patients without renal failure (NRF). The size distribution study showed significant difference in EVs mode diameter between CD and UD. Turco et al. [26] showed that decreases of EVs may reflect atherosclerosis and thrombosis-related activity in renal capillaries and parenchyma.

Currently kidney function is monitored by measuring serum creatinine, creatinine clearance, and proteinuria. These clinical markers are usually a late sign of renal damage and indicate its dysfunction [6]. What is more, these markers do not always correlate well with the severity of renal damage seen on biopsy [27]. The early stages of renal functions impairment are diagnosed only by measuring GFR. The complications of chronic renal disease increase with decreasing GFR [28]. There are a number of studies confirming the huge impact of GFR level in progression of renal damage [29–32]. However, a good biomarker of decreased GFR, together with a proper marker of tubular injury, would allow for the diagnosis of renal failure in diabetic patients before increased albuminuria and irreversible kidney damage [13].

One of the specific renal proteins—uromodulin—has been found as urinary biomarker which positively correlates with GFR ratio and decreased uromodulin concentrations have been found in renal failure and diabetic nephropathy [33, 34]. In our study, we observed that uromodulin is a prominent protein related to EVs and we may assume that its drop in urine concentrations is related to decreased EV density in patient with renal failure. Uromodulin is probably involved in EVs clustering and precipitation (Figure 1(c)) [35].

In our study, we observed a negative correlation between EVs density and serum glucose level in RF and a negative tendency between these parameters in UD. Mehta [36] showed that the kidney is intimately involved in the development of hyperglycemia in the critically ill patients. Sechi et al. [37] demonstrated that abnormal plasma glucose levels were elevated when the GFR was <50 mL/min/1.73 m^2^ and overall glucose metabolism parameters were not correlated with microalbuminuria (MA). These data are consistent with our study. We did not observe the correlation between MA and the EVs density in both CD and UD, as well as in RF and NRF. Thus we may assume that EVs presence is more related with impaired glucose metabolism and then with the presence of renal damage biomarker (MA) and EVs can be treated as the more ominous label of disease in diabetic patients. Interestingly, we also observed a positive correlation between EVs density and urine creatinine concentration in NRF and CD, in contrast to those with more advanced disease stage (RF) or impropriate treatment (UD). This observation may suggest that the early renal dysfunction processes are more considerable in the urine EVs release. In the milder stage of renal failure we may expect the higher number of EVs in urine, as the primary marker of the renal dysfunction. In further study the more specific attention should be focused on the correlation between cystatin C and even angiopoietin 2, which appeared to be a relevant predictor of renal dysfunction in acute pancreatitis patients [38]. Age is the strongest factor influencing physiological state, as well as renal function. According to the number of epidemiological studies, GFR declines with age average about 0.8 mL/min/1.73 m^2^ per year. The multiple regression (backward stepwise regression) model did not show the significant impact of age on changes in the amount of EVs.

Urinary EVs are enriched in membrane and cytosolic cargo proteins from the different epithelial cells lining the urinary track. To date, there are only few studies to reveal the new urine biomarkers, which are based on proteomic methods. Among them, proteases and protease inhibitors including kallikreins [6, 10, 14] and metalloproteinases (MMP-2, MMP-7, and MMP-8) have been observed in normoalbuminuria and microalbuminuria groups. In macroalbuminuric patients, cathepsin D has been more abundant than in other patients [14]. In our study we found new protease inhibitors including inter-alpha-trypsin inhibitor (AMBP-Human), inhibitor of the complement membrane attack complex (CD59) and proteases including mannan-binding lectin serine protease 2 (MASP2_Human). A large number of unique proteins in controlled diabetes can be used in selection of potential biomarkers in further studies on the risk of diabetic nephropathy in such patients.

According to our observations the specific marker for exosomes (CD81) did not show significant difference in the level (Figure 5). The CD81 is a surface exosome marker which belongs to the tetraspanin family (TAPA-1) and is involved in signal transduction and cell adhesion [39]. CD81 protein is enriched in the exosome membrane [40]. However, it has not been shown if this biomolecule distinguish the severity of renal disease in diabetic patients. We may also speculate that in our study we did not observe significant differences in CD81 level between groups because the most numerous was CD81-negative (nonexosomes) population of microvesicles, membrane bubbles above 100 nm (Figures 1(e) and 1(f)).

In our study we confirmed that the quantitative analysis of urinary EVs seems to be a promising tool for defining new biomarkers [41]. We pictured that EVs are present in urine and they maintain their integrity during sampling and preparation process. However, most of these studies are focused on the two areas of progress: bladder or prostatic cancer and acute rejection of renal transplant [10]. Very recent study on diabetic nephropathy novel biomarkers revealed that exosomal regucalcin was underexpressed in renal disease patients [42].

## 5. Conclusions

Finally we may conclude that urinary EVs have the potential to be biomarkers of renal damage in diabetic patients, and the number of structural and enzymatic proteins (including uromodulin) can be found in urine EVs fraction to be in use as their indicators or new biomarkers both of renal failure and of diabetic nephropathy in the future. The easy accessibility of EVs in urine can increase their use as biomarkers compared to invasive biopsy. Further validation, characterization of the content of bioactive molecules, and larger study population are needed.

## Supplementary Material

Supplementary Table 1 presents the results of backward stepwise regression analysis to identify the impact of independent predictors (age, serum glucose, urine creatinine and albumin) on the EVs density (number of extracellular vesicles per milliliter; n/mL) in the control group.
Supplementary Table 2 presents the results of nonparametrical regression analysis (the Spearman test) to identify the relationships between an epidemiological parameter (age) and selected biochemical biomarkers (serum glucose, urine creatinine and albumin, serum creatinine, GFR and EVs diameter and density) in study groups.
Abbreviations: EVs - Extracellular vesicles; GFR - Glomerular Filtration Rate.

## Figures and Tables

**Table 1:** Clinical characteristics, blood, and urine biochemistry of study groups: C, CD, and UD.

	C *n* = 10	CD *n* = 24	UD *n* = 36	*p* value
Age (years)	52 (7)	62 (15)^†^	61 (12)^†^	0.0683
Gender (male/female)	6/4	17/7	23/13	—
Serum glucose	5.2	6.8^†**∗**^	9^†**∗**^	**<0.0001**
(mmol/L)	(5.0–5.5)	(5.9–7.9)	(7.4–12)	
Urine albumin	6	6^**∗**^	37^†**∗**^	**<0.0001**
(mg/L)	(4–13)	(2–22)	(12–267)	
Urine creatinine	15	5^†^	7^†^	**0.0054**
(mmol/L)	(9–17)	(4–9)	(5–11)	
Serum creatinine (*µ*mol/L)	72 (60–85)	77 (67–98)	79 (62–108)	0.4696
GFR (mL/min/1.73 m^2^)	87 (76–101)	77 (59–95)	79 (59–97)	0.5114
EVs density	5.2*E*10	8.4*E*10	5.2*E*10	0.5013
(number/mL)	(2.7*E*10–1.9*E*11)	(3.9*E*10–1.7*E*11)	(2.6*E*10–1.5*E*11)	
EVs mode diameter	106	115^†**∗**^	109^**∗**^	**0.0212**
(nm)	(104–110)	(107–118)	(106–112)	
EVs mean diameter (nm)	123 (4)	134 (11)^†^	129 (8)	**0.0065**

^†^Significant in comparison with the control group at *p* < 0.05.

^*∗*^Significant difference between subgroups CD and UD at *p* < 0.05.

Bold means statistically significant difference between the three groups at *p* < 0.05.

**Table 2:** Clinical characteristics, blood, and urine biochemistry of study groups: C, RF, and NRF.

	C *n* = 10	RF *N* = 15	NRF *N* = 45	*p* value
Age (years)	52 (7)	69 (11)^†**∗**^	60 (3)^†**∗**^	**0.0027**
Gender (male/female)	6/4	15/3	25/17	—
Serum glucose	5.2	8.7^†^	7.9^†^	**<0.0001**
(mmol/L)	(5.0–5.5)	(6.5–11)	(6.5–10)	
Urine albumin	6^†^	51^†^	14	0.0923
(mg/L)	(4–13)	(7–359)	(4–58)	
Urine creatinine	15	6^†^	8^†^	**0.0046**
(mmol/L)	(9–17)	(5–8)	(4–11)	
Serum creatinine	72	119^†**∗**^	73^**∗**^	**<0.0001**
(*µ*mol/L)	(60–85)	(111–123)	(60–84)	
GFR	87	49^†**∗**^	89^**∗**^	**<0.0001**
(mL/min/1.73 m^2^)	(76–101)	(39–55)	(73–105)	
EVs density	5.2*E*10	2.6*E*10^**∗**^	8.7*E*10^**∗**^	**0.0361**
(number/mL)	(2.7*E*10–1.9*E*11)	(2.0*E*10–8.2*E*10)	(4.0*E*10–1.9*E*11)	
EVs mode diameter (nm)	106 (104–110)	111 (105–115)	109 (107–115)	0.1965
EVs mean diameter	122	129	129^†^	**0.0101**
(nm)	(120–126)	(123–138)	(126–136)	

^†^Significant in comparison with the control group at *p* < 0.05.

^*∗*^Significant difference between subgroups RF and NRF at *p* < 0.05.

Bold means statistically significant difference between the three groups at *p* < 0.05.

**Table 3:** Results of Spearman's *rho* test for correlations between EVs density and biochemical parameters.

	C *n* = 10	CD *n* = 24	UD *n* = 36	RF *n* = 15	NRF *n* = 45
Serum glucose	0.49	−0.27	−0.33	**−0.66**	−0.21
(mmol/L)	*p* = 0.15	*p* = 0.19	*p* = 0.05	**p = 0.01**	*p* = 0.16
Urine creatinine	0.08	**0.52**	0.03	−0.46	**0.33**
(mmol/L)	*p* = 0.83	**p = 0.01**	*p* = 0.87	*p* = 0.08	**p = 0.03**
Urine albumin	−0.16	0.37	0.14	−0.03	0.25
(mg/L)	*p* = 0.65	*p* = 0.08	*p* = 0.42	*p* = 0.92	*p* = 0.09
GFR	0.50	0.26	0.27	**−0.54**	0.07
(mL/min/1.73 m^2^)	*p* = 0.14	*p* = 0.21	*p* = 0.12	**p = 0.04**	*p* = 0.66

Bold means statistically significant correlation at *p* < 0.05 level.

## Abbreviations

CD: Properly controlled diabetic patients
DMt1: Type 1 diabetes mellitus
DMt2: Type 2 diabetes mellitus
EDTA: Ethylenediaminetetraacetic acid
ESEM: Environmental Scanning Electron Microscopy
EVs: Extracellular vesicles
GFR: Glomerular Filtration Rate
GO: Gene Ontology
HbA1c: Glycated hemoglobin
MA: Microalbuminuria
NRF: Diabetic patients without renal failure
PBS: Phosphate-buffered saline
RF: Diabetic patients with renal failure
TEM: Transmission Electron Microscopy
TRPS: Tunable Resistive Pulse Sensing
UD: Poorly controlled diabetic patients.
