# Supplementary material for: Urinary Extracellular Vesicles: Potential Biomarkers of Renal Function in Diabetic Patients
Source: J Diabetes Res. 2016 Dec 25;2016:5741518. doi: 10.1155/2016/5741518 (PMC5220476; doi:10.1155/2016/5741518)
Supplement: Supplementary file 1 — Supplementary Table 1 presents the results of backward stepwise regression analysis to identify the impact of independent predictors (age, serum glucose, urine creatinine and albumin) on the EVs density (number of extracellular vesicles per milliliter; n/mL) in the control group. Supplementary Table 2 presents the results of nonparametrical regression analysis (the Spearman test) to identify the relationships between an epidemiological parameter (age) and selected biochemical biomarkers (serum glucose, urine creatinine and albumin, serum creatinine, GFR and EVs diameter and density) in study groups. Abbreviations: EVs - Extracellular vesicles; GFR - Glomerular Filtration Rate. [file 5741518.f1.docx]

|  |  |  |  |  |  |
| --- | --- | --- | --- | --- | --- |

**Supplementary Table 1. Results of backward stepwise regression - EVs density vs age, serum glucose, urine creatinine, albumin creatinine in control group.**

| **Step I** | | | | | |
| --- | --- | --- | --- | --- | --- |
|  | **B** | **Std. Error** | **Beta** | **t(7)** | **Sig.** |
| **Free term** |  |  | -1,214272E+11 | -0,23557 | 0,820513 |
| **Serum glucose** | -0,086710 | 0,389607 | -2,526921E+10 | -0,22256 | 0,830235 |
| **Urine creatinine** | 0,331293 | 0,326026 | 1,063119E+10 | 1,01615 | 0,343385 |
| **Age** | 0,432672 | 0,384899 | 5,819337E+09 | 1,12412 | 0,298029 |
| **Urine albumin** | -0,434282 | 0,390284 | -1,794182E+09 | -1,11273 | 0,302570 |
| **Step II** | | | | | |
|  | **B** | **Std. Error** | **Beta** | **t(8)** | **Sig.** |
| **Free term** |  |  | -2,250526E+11 | -1,08402 | 0,309941 |
| **Urine creatinine** | 0,301410 | 1,022743E+10 | 9,672266E+09 | 0,321212 | 0,252731 |
| **Age** | 0,318064 | 5,272751E+09 | 4,277883E+09 | 0,252731 | 0,321212 |
| **Urine albumin** | 0,306037 | -1,596903E+09 | 1,264354E+09 | 0,242149 | 0,242149 |
| **Step III** | | | | | |
|  | **B** | **Std. Error** | **Beta** | **t(9)** | **Sig.** |
| **Free term** |  |  | -1,581368E+11 | -0,79457 | 0,447309 |
| **Age** | 0,484510 | 0,307804 | 6,516537E+09 | 1,57409 | 0,149919 |
| **Urine albumin** | -0,373902 | 0,307804 | -1,544728E+09 | -1,21474 | 0,255372 |
| Stage IV | | | | | |
|  | **B** | **Std. Error** | **Beta** | **t(10)** | **Sig.** |
| **Free term** |  |  | -1,003773E+11 | -0,507467 | 0,622831 |
| **Age** | 0,355219 | 0,295604 | 4,777616E+09 | 1,201672 | 0,257176 |

**Supplementary Table 2. Results of Spearman’s test for correlation between age and biochemical parameters in study groups.**

| **Control** | | |
| --- | --- | --- |
|  | **rho** | **p** |
| **Age vs serum glucose** | 0.15 | **0.65** |
| **Age vs urine creatinine** | 0.18 | **0.58** |
| **Age vs urine albumin** | -0.09 | **0.77** |
| **Age vs serum creatinine** | -0.21 | **0.5** |
| **Age vs GFR** | 0.03 | **0.92** |
| **Age vs EVs mode diameter** | -0.4 | **0.19** |
| **Age vs EVs mean diameter** | -0.1 | **0.75** |
| **Age vs EVs density** | 0.07 | **0.82** |
| **CD** | | |
| **Age vs serum glucose** | 0.06 | 0.77 |
| **Age vs urine creatinine** | -0.37 | 0.08 |
| **Age vs urine albumin** | -0.12 | 0.57 |
| **Age vs serum creatinine** | 0.33 | 0.11 |
| **Age vs GFR** | -0.58 | 0.03 |
| **Age vs EVs mode diameter** | 0.25 | 0.24 |
| **Age vs EVs mean diameter** | 0.09 | 0.66 |
| **Age vs EVs density** | -0.23 | 0.28 |
| **UD** | | |
| **Age vs serum glucose** | -0.22 | 0.18 |
| **Age vs urine creatinine** | -0.32 | 0.06 |
| **Age vs urine albumin** | 0.16 | 0.35 |
| **Age vs serum creatinine** | 0.29 | 0.09 |
| **Age vs GFR** | -0.3 | 0.07 |
| **Age vs EVs mode diameter** | -0.06 | 0.73 |
| **Age vs EVs mean diameter** | 0.1 | 0.56 |
| **Age vs EVs density** | -0.13 | 0.46 |
| **RF** | | |
| **Age vs serum glucose** | -0.17 | 0.55 |
| **Age vs urine creatinine** | -0.07 | 0.81 |
| **Age vs urine albumin** | -0.61 | 0.02 |
| **Age vs serum creatinine** | -0.08 | 0.76 |
| **Age vs GFR** | -0.15 | 0.59 |
| **Age vs EVs mode diameter** | -0.3 | 0.28 |
| **Age vs EVs mean diameter** | -0.03 | 0.93 |
| **Age vs EVs density** | 0.11 | 0.68 |
| **NRF** | | |
| **Age vs serum glucose** | -0.18 | 0.24 |
| **Age vs urine creatinine** | -0.35 | 0.02 |
| **Age vs urine albumin** | 0.14 | 0.37 |
| **Age vs serum creatinine** | 0.07 | 0.66 |
| **Age vs GFR** | -0.23 | 0.13 |
| **Age vs EVs mode diameter** | 0.24 | 0.11 |
| **Age vs EVs mean diameter** | 0.18 | 0.25 |
| **Age vs EVs density** | -0.09 | 0.55 |
